# Duplicate Submission, Zero Consequences: A Reviewer’s First-Person Case Study

**DOI:** 10.7759/cureus.99518

**Published:** 2025-12-18

**Authors:** Enzo Emanuele

**Affiliations:** 1 Pathology, 2E Science, Robbio, ITA

**Keywords:** academic publishing, duplicate submission, peer review integrity, publication ethics, research misconduct, risk-reward asymmetry, simultaneous submission

## Abstract

Although submitting identical or substantially overlapping manuscripts to multiple journals constitutes research misconduct, current detection mechanisms rely mostly on chance. This creates a risk-reward landscape in which unethical authors may face minimal consequences even when duplicate submissions are identified, while gaining a considerable advantage when they are not. This case study presents the firsthand discovery of a duplicate submission during peer review and describes major shortcomings in the subsequent editorial handling. On April 28, 2025, while reviewing a manuscript on occupational radiation exposure for an academic journal, a routine literature search showed that a nearly identical article had been published earlier that month in another open-access journal. Despite prompt notification from the editorial office, the journal continued with standard peer review, obtaining four full referee reports before rejecting the submission on May 28, 2025. The decision letter made no mention of an ethics investigation, institutional notification, or any corrective action. The authors appear to have faced no meaningful sanctions, having already published the article in another journal while the same manuscript remained under review elsewhere.

This case illustrates how unethical authors can submit the same work to multiple journals simultaneously, wait for the fastest or most favorable review, and abandon other submissions without penalty. At worst, they receive a routine rejection without ethical consequences. These observations suggest that penalties for detected duplicate submissions are minimal, whereas the potential benefits of undetected misconduct remain high. To correct this incentive imbalance, future digital infrastructure initiatives in scholarly publishing should establish robust pre-submission and pre-review screening protocols, standardized investigation procedures for suspected violations, and enforceable sanctions, including institutional notification, time-limited submission bans, and, most importantly, the retraction of published articles found to have been the subject of duplicate submission.

## Editorial

Duplicate or simultaneous submission, defined as submitting substantially similar or identical manuscripts to multiple journals at the same time, is widely recognized as research misconduct and explicitly prohibited by major editorial guidelines [1-3]. The International Committee of Medical Journal Editors (ICMJE) states unequivocally that authors should not submit the same manuscript to more than one journal concurrently, while the Committee on Publication Ethics (COPE) considers simultaneous submission a clear breach of publication ethics requiring investigation [4]. Beyond breaching formal policies, this practice undermines the fundamental assumptions of exclusivity and novelty that underpin editorial decision-making and fair competition among journals [4,5].

The harms of duplicate submission are both practical and structural. Most immediately, duplicate submission wastes scarce peer-review capacity by prompting multiple sets of reviewers to evaluate the same work, exacerbating well-documented concerns about reviewer fatigue and the sustainability of peer review [6,7]. Accordingly, rates of agreement to peer review, review completion rates, and timeliness of reviewer responses have declined significantly over the last five years [8]. Of note, each hour a reviewer spends evaluating a duplicated manuscript represents time unavailable for reviewing legitimate original work [1-3]. Beyond resource waste, this practice may also erode trust in the editorial process, as editors base decisions on the expectation that they are considering a unique contribution rather than competing unknowingly with parallel submissions elsewhere [5].

Despite routine author attestations that manuscripts are not under consideration elsewhere, verification mechanisms remain largely inadequate, and detection of duplicate submissions typically relies on chance rather than systematic screening. Notably, while considerable resources have been devoted to plagiarism detection tools [9], image manipulation screening [10], and paper mill identification [11], cross-publisher detection of simultaneous submissions remains unavailable. For unethical authors, this environment creates a highly asymmetric risk-reward dynamic in which undetected duplicate submission can accelerate publication and provide a competitive edge. While detection generally can simply result in a standard rejection, no different from the outcome of sequential, legitimate submissions when publication standards are not met. This case study describes the discovery of an apparent duplicate submission during peer review and illustrates how weak detection mechanisms, combined with suboptimal editorial handling and minimal consequences, can maintain a risk-reward structure that makes duplicate submission a rational, albeit unethical, strategy under current enforcement conditions.

In April 2025, I received an invitation through Editorial Manager to review a manuscript for a mid-tier, PubMed-indexed occupational medicine journal employing double-blind peer review. The manuscript concerned occupational radiation exposure among healthcare workers, a topic within my expertise. I accepted the invitation and accessed the full manuscript, hereafter designated Manuscript A to maintain confidentiality. Following my standard practice, I began with a literature search to contextualize the work, assess citation adequacy, evaluate novelty, and judge whether conclusions were in line with existing knowledge. Using PubMed and Scopus with keywords related to occupational radiation exposure, I discovered within the first hour a paper published two weeks earlier by a large open-access publisher (hereafter Article B) with an identical title to Manuscript A. Notably, Article B’s abstract described an identical study design, including the same data collection period, same healthcare facility type, same occupational groups monitored, and same research protocol. The stated objectives, though slightly reworded, addressed precisely the same research questions. Most tellingly, the reported sample size matched exactly, a highly improbable coincidence for independent studies.

I subsequently conducted a detailed section-by-section comparison of both documents (Table 1). The introduction sections covered identical background literature with substantial citation and argumentation overlap, though with minor rewording (possibly reflecting peer-review requests for Article B). The methods sections described identical study populations, inclusion/exclusion criteria, data collection procedures, and statistical analytical approaches. Both manuscripts cited the same institutional review board approval number, confirming they derived from the same approved research protocol. The results section provided unambiguous evidence of duplication. Every numerical value in Manuscript A’s tables appeared in Article B’s tables with exact matches to multiple decimal places. All sample sizes across subgroups were identical. All statistical test results, including p-values, matched numerically. The discussion sections showed extensive textual similarity, with strategic rewording likely reflecting peer review revisions incorporated into Article B. Approximately 80% to 90% of the discussion text was either verbatim identical or minimally paraphrased through synonym substitution and sentence restructuring while preserving substantive content and argumentation flow.

**Table 1:** Comparison of key elements between manuscript A and article B IRB: Institutional Review Board

Element	Degree of overlap	Details
Title	Identical	Word-for-word match
Study design	Identical	Same time period, facility type, occupational groups, monitoring practices
Sample size	Identical	Exact numerical match across all subgroups
IRB approval	Identical	Same approval number cited in both
Numerical results	100% identical	All sample sizes, dose measurements, statistical test values, and p-values matched to multiple decimal places
Methods section	Near-identical	Same inclusion/exclusion criteria, data collection procedures, statistical methods
Discussion text	80% to 90% overlap	Mix of verbatim sentences and minimal paraphrasing
Citations	Substantial overlap	Same reference list with minor additions/deletions

The timeline analysis was particularly revealing. Article B was published online in mid-April 2025 with a digital object identifier (DOI), while Manuscript A was still under active review at the journal where I was reviewing. The open-access publisher’s website indicated Article B had been received in September 2024 and accepted in April 2025 following peer review. This timeline suggested two plausible scenarios, both representing clear ethics violations. First, the authors may have submitted to both journals simultaneously in September 2024, with the open-access journal completing review first while the second journal progressed more slowly. Second, the authors may have submitted Manuscript A to the second journal after receiving a revision request from the open-access publisher but before final acceptance, essentially hedging against potential rejection at the first venue. Under either scenario, the authors submitted identical work to at least two different journals while presumably signing declarations at both venues attesting the work was not under consideration elsewhere, a major breach of publication ethics.

I immediately suspended detailed scientific review, as evaluating already-published material submitted in apparent violation of ethics policies served no useful purpose. Within 24 hours of discovering Article B, I submitted a review report to the journal editors documenting my findings comprehensively. This communication included Article B’s full citation and DOI, a detailed point-by-point comparison highlighting identical sample sizes and numerical results, documentation of substantial textual overlap in discussion sections, and a note of the identical IRB approval number confirming common origin. I recommended immediate rejection without completing peer review and referral for formal misconduct investigation in accordance with the Committee on Publication Ethics (COPE) guidelines. Surprisingly, the editorial office did not halt the review process upon receiving this report. Through the manuscript tracking system visible to reviewers, I observed that the manuscript remained in active review status. Eventually, four complete reviews were submitted, including my own report. The other three reviewers conducted standard scientific reviews without discovering the prior publication, offering typical constructive criticism about methodological details, interpretation caveats, and presentation clarity.

In late May 2025, approximately one month after my review submission, the journal rejected Manuscript A. However, the rejection letter employed standard boilerplate language providing no indication that an ethics violation had been identified or would carry consequences beyond this single manuscript decision. In addition, I received no follow-up communication indicating what investigation, if any, had been conducted. I was not informed whether the authors had been asked to explain the relationship between their submission and Article B, whether they provided any justification or acknowledgment, or whether the matter had been referred to COPE or the authors’ institutions for investigation. The editorial correspondence suggested the manuscript had been rejected through normal peer review based on aggregated reviewer feedback, with the duplicate submission treated as one among several substantive concerns rather than as a categorical disqualification requiring special handling and investigation.

Article B is currently published without a notation about the duplicate submission attempt to another journal. As a reviewer who invested several hours evaluating a paper already published two weeks before I received the review invitation, I do not know whether the authors faced any consequences beyond standard rejection at the second journal, such as submission bans, formal warnings, or institutional notification that would enable research integrity office investigation. Most critically, without institutional notification, their research integrity offices cannot address potential systemic problems in research conduct oversight or implement corrective measures to prevent recurrence.

This case offers direct evidence of the asymmetric risk-reward system that renders duplicate submission a rational, though highly unethical, option under current enforcement practices. Table 2 summarizes the outcomes and consequences for unethical authors across three submission scenarios.

**Table 2:** Potential outcomes of duplicate submissions

Scenario	Time to publication	Reviewer burden	Author consequences	Net author benefit
Compliant sequential submission	Six to 24 months (depending on rejections)	Standard single-journal process	None	Publication after an extended timeline
Successful duplicate submission (undetected)	Three to six months (parallel processing)	Doubled burden across journals	None	Publication with substantial time savings
Detected duplicate submission (current study)	Three to six months (Article B published while Manuscript A was rejected)	Wasted effort	Rejection from one journal only; no investigation, no institutional notification, no future consequences	Publication in Article B with time savings; same outcome as sequential submission, but faster

As Table 2 shows, detection currently carries little downside for unethical authors, whereas successful evasion offers clear advantages. Detection in this case led only to a routine rejection at one journal, while the authors still achieved publication elsewhere without investigation or institutional notification. Simultaneous submission effectively turned the second journal into a backup venue; absent detection, the authors could simply choose the journal offering the fastest or most favorable review and disregard the other’s revision requests, or at worst accept a standard rejection. Under such conditions, the expected costs of misconduct do not exceed the expected benefits, undermining deterrence. Decision theory reasoning [12] therefore predicts that rational but unethical authors will favor duplicate submission when detection probability is low, especially given that sequential resubmission after rejection can defer publication by one to two years for researchers under pressure from tenure clocks, grant deadlines, and funding timelines [13].​

Although nearly all journals prohibit duplicate submission and require attestations, current verification systems remain weak, with detection largely dependent on chance rather than systematic checks. In this case, the duplication surfaced only because of unusually thorough literature searching, which is not uniformly practiced and could easily have failed had different search terms been used or the title been strategically altered. The papers were characterized by identical titles and were submitted to journals indexed in major databases; however, more subtle strategies, including different titles or substantially reworded abstracts, could readily have escaped notice. Effective detection at scale would require shared databases of manuscripts under review, a solution that may raise legitimate concerns about confidentiality and cross-publisher cooperation but currently represents the only realistic technological path to systematic screening. In the absence of such infrastructure, journals depend on easily circumvented honor-based declarations that manuscripts are not under consideration elsewhere.​

Investigation failures further weaken enforcement. Despite clear evidence of misconduct in this case study, including identical numerical data, extensive textual overlap, and matching IRB approval numbers, the journal at which I was reviewing continued routine peer review instead of pausing evaluation and initiating a formal investigation consistent with COPE guidelines [4]. Treating detailed misconduct reports as ordinary reviewer comments rather than categorical disqualifications suggests that some journals either lack robust protocols or choose to avoid the administrative and legal burdens of full investigations, defaulting to simple rejection as the least demanding option. This approach not only allows misconduct to proceed largely unchallenged but also signals that ethical breaches of duplicate submission will seldom trigger serious scrutiny.​ Continuing review after receiving documented evidence of duplication clearly wastes scarce reviewer capacity [14] and exacerbates reviewer fatigue or dissatisfaction [15]. In this case, other reviewers continued their evaluation of Manuscript A even after duplication had been documented, diverting effort that could have supported other submissions and further straining an already fragile peer review ecosystem. The journal also forfeited an opportunity for accountability; the authors received a generic rejection notice with no indication that their behavior had been recognized as an ethics violation or would influence future submissions. Unlike other forms of misconduct, which are often addressed through retractions, public notices, or documented image manipulation findings [16], duplicate submission currently lacks comparable transparency mechanisms, leaving institutions, other journals, and the broader community unaware of violations.​

Consequence failures complete the incentive problem. In the absence of meaningful sanctions, duplicate submission remains a low-risk, high-reward tactic. Proportionate consequences could include temporary or permanent submission bans, formal notification of institutional research integrity offices, publisher-level databases documenting violations, and retraction of the published article when a duplicate submission is detected. In addition, formal institutional disclosure of confirmed violations, including documented communication to the author's institution and, when appropriate, public notification through journals' ethics sections or publisher-maintained registries, would create reputational and career consequences that reinforce deterrence. Such transparent accountability mechanisms can counterbalance the perceived benefits of misconduct by ensuring that violators face lasting professional consequences beyond simple rejection. None of these measures was applied in the case I am reporting; the authors experienced only a routine rejection at one journal while their article proceeded to be published and unmarked at another, reinforcing the perception that such misconduct is unlikely to attract penalties beyond the outcome of ordinary peer review.

From a systemic perspective, the true prevalence of duplicate submissions cannot be reliably estimated because detection remains sporadic and unsystematic, as this first-person account demonstrates. The current incident almost certainly represents a visible instance of a much larger, mostly hidden problem, given the proliferation of journals, partially overlapping indexing, and fragmented reviewer pools that lower the probability of independent detection. At the same time, structural incentives within academic careers, such as an emphasis on publication counts, impact metrics, and rapid output, encourage strategies that shorten time to publication [13]. When combined with weak detection, inconsistent investigation, and negligible consequences, these pressures create conditions in which ethically questionable shortcuts become appealing for some authors.​

Addressing these asymmetric incentives requires coordinated reforms that increase detection, strengthen consequences, and reduce the perceived benefits of misconduct. First, detection must become more systematic. Ideally, publishers should develop tools to cross-check new submissions against databases of manuscripts under review. Second, consequences must be compelling and consistently applied. Upon receipt of credible evidence, journals should suspend review, initiate a formal investigation involving author response, cross-journal coordination, and consultation with ethics frameworks such as COPE, and ensure that confirmed violations trigger institutional notification, time-bound submission bans, publisher-level documentation, and retraction of any published article related to the duplicated submission. Third, the benefits of gaming the system should be reduced by accelerating and standardizing legitimate editorial timelines, setting and honoring target decision windows, and improving status transparency, while institutions shift from output-focused metrics toward evaluations that emphasize rigor, quality, and integrity.​

This case study should be interpreted in the context of several limitations. As a single-case observation, it cannot quantify the prevalence of duplicate submissions or characterize typical journal responses across the literature. The journal’s handling may not reflect wider practice, and some internal deliberations may have occurred without communication to the reporting reviewer, although the lack of visible action suggests any investigation was limited at best. The authors’ motivations and potential alternative explanations cannot be definitively established, but the convergence of identical data, matching IRB approvals, and timeline reconstruction strongly favors a scenario of simultaneous or overlapping submission, and no alternative account was provided despite detailed documentation.

In summary, this first-person case study exemplifies critical enforcement deficiencies that render duplicate submission a rational, albeit ethically unsound, strategy under prevailing circumstances. Three principal failures sustain this issue. First, the detection of duplicate submissions remains predominantly serendipitous, rather than the product of systematic surveillance. Second, journals frequently lack structured investigative protocols and tend to default to perfunctory rejection when formal procedures are lacking. Third, the absence of substantive consequences means that violators incur no penalties beyond those experienced by legitimate authors following standard rejection. Remediation of these deficiencies necessitates coordinated, sector-wide interventions. In this scenario, publishers should establish robust cross-journal screening mechanisms, while journals must implement compulsory, procedure-driven investigations upon identification or reporting of suspected violations. Upon confirmation of a duplicated submission, violators should face substantive, enforceable sanctions, including institutional notification, submission bans, and retraction of published articles. Absent these reforms, duplicate submission will persist as a low-risk, high-reward behavior, one that squanders peer reviewer effort, erodes scientific integrity, and incentivizes publication misconduct.
